# Polymicrobial-driven NLRP6 inflammasome regulates IL-1β production and alveolar bone loss in a murine model of periodontitis

**DOI:** 10.1371/journal.ppat.1014574

**Published:** 2026-09-15

**Authors:** Sarah Metcalfe, Rajendra P. Settem, Edwin J. Ovalle, Michelle Panasiewicz, Alejandro Escobar, Jason G. Kay

**Affiliations:** 1 Department of Oral Biology, School of Dental Medicine, University at Buffalo, Buffalo, New York, United States of America; 2 Institute for Research in Dental Sciences, Faculty of Dentistry, Universidad de Chile, Santiago, Chile; 3 Department of Microbiology and Immunology, Jacobs School of Medicine and Biomedical Sciences, University at Buffalo, Buffalo, New York, United States of America; Lunds universitet Medicinska fakulteten, SWEDEN

## Abstract

Periodontal disease is a chronic inflammatory condition that develops in response to oral microbiome dysbiosis and host-microbiome immune response dysregulation. The innate immune system plays a major role in the development and persistence of disease, in part by producing inflammatory cytokines. One of the major cytokines implicated in disease is interleukin-1β (IL-1β), which requires inflammasome activation. Much of the oral microbiome, including Streptococci, which are otherwise considered commensal, is required for the full development of periodontal disease. We have previously reported that inflammatory-activated macrophages and neutrophils counterintuitively allow survival of internalized *Streptococcus gordonii* over non-activated phagocytes. This internal bacterial survival leads to inflammasome activation via the cytoplasmic activator NLRP6, but not NLRP3, and subsequent increases in IL-1β release. Here, we test and find that the keystone pathogen *Porphyromonas gingivalis* can activate macrophages in a manner that allows for increased *S. gordonii* survival and IL-1β production above levels when *P. gingivalis* interacts with macrophages alone. We also use the mouse ligature-induced periodontal disease model to test the importance of NLRP6 in disease development. We found mice lacking NLRP6 had significantly reduced bone loss, IL-1β, and neutrophil infiltration following disease induced by *P. gingivalis* when *S. gordonii* or other mouse commensals were present, but had no effect when *S. gordonii* was inoculated alone. This work thus reveals an additional important inflammasome activation mechanism by which oral keystone pathogens may stimulate periodontal disease progression.

## Author summary

Chronic inflammation is a driver of many diseases, including periodontal disease. Periodontal disease is a long-lasting inflammatory disease caused by an unhealthy imbalance in the oral microbiome and an abnormal immune response toward those bacteria. A major inflammatory molecule involved in this inflammation is IL-1β, which is produced after inflammasome activation. We previously found that immune cells such as macrophages and neutrophils can unexpectedly allow *Streptococcus gordonii*, a normally health-associated oral bacterium, to survive within the immune cells and to trigger the NLRP6 inflammasome, leading to increased IL-1β release. In this study, we found that *Porphyromonas gingivalis*, a key periodontal pathogen, stimulates macrophages to allow *S. gordonii* survival and increased IL-1β production. Using a mouse model of periodontal disease, we also found that without NLRP6 mice had less bone loss, less inflammation, and fewer neutrophils when *P. gingivalis* was present along with other oral bacteria. These results show that NLRP6 plays an important role in how oral bacteria work together to worsen periodontal disease.

## Introduction

Periodontal disease is the most common chronic inflammatory condition not caused by a single organism, but through the development of a dysbiotic, or imbalanced, community of microorganisms. Dysbiotic oral microbiomes include some normally commensal organisms, along with keystone pathogens that are capable of driving this dysbiosis as minority members [1–5]. *Porphyromonas gingivalis* is the most well-characterized keystone pathogen in periodontal disease development. *P. gingivalis* has the ability to inhibit some cellular signaling of, and survive within, immune cells such as dendritic cells and macrophages [6–12]. This disruption of inflammatory response contributes to the emergence of bacterial dysbiosis and a resultant positive feedback loop of inflammation leading to more dysbiosis, and ultimately the development of periodontal disease [2,13,14]. Periodontal disease is not caused solely by keystone pathogens, but also involves opportunistic bacteria, including normally commensal species that contribute under dysbiotic conditions [2]. Streptococci are part of the normal oral flora; however, they can also colonize extra-oral sites and contribute to systemic disease [15–19]. *Streptococcus gordonii*, a commonly studied model oral streptococcus, can promote periodontitis by assisting *P. gingivalis* colonization and growth, thereby acting as an accessory pathogen to enhance the pathogenicity of *P. gingivalis* [20–22]. In addition, oral streptococci, while generally associated with the healthy oral microbiome due to them making up a large percentage of the microbiome, are present in higher absolute numbers during gingivitis and periodontal disease, even though they make up a lower percentage of the overall microbiome [5,23–25].

Macrophages are an important cell type in the oral cavity, playing essential roles in maintaining mucosal immunity, including tolerance, and tissue homeostasis by physically interacting with and responding to oral microbes [26–30]. In gingivitis and periodontal disease, the number of inflammatory or classically activated (M1) macrophages in the oral cavity increases, along with the inflammatory immune response they promote [31–34]. Indeed, depletion of macrophages reduces alveolar bone resorption by modulating the host immune response [35], and recruitment of non-activated or alternatively activated (M0 or M2) macrophages by CCL2, reduces alveolar bone loss in mouse models of periodontitis [36].

As part of the inflammatory response, macrophages produce cytokines and chemokines to orchestrate the immune response [30,37]. One such cytokine, IL-1β, is produced in an inflammasome-dependent manner and promotes inflammation, stimulates fever, and recruits and activates other immune cells [37,38]. Greatly increased IL-1β occurs in gingivitis and chronic periodontitis [33,39,40], and IL-1β has emerged as a possible therapeutic target in periodontal disease and other chronic inflammatory diseases [41–44]. Contributing to periodontal disease-associated inflammation are infiltrating inflammatory macrophages, which have increased inflammasome components along with high levels of IL-1β production that lead to further promotion of inflammation and alveolar bone resorption [32,33,39,41,45].

We have found that *S. gordonii* is better able to survive within and damage the phagosomes of inflammatory macrophages over non-activated macrophages, due to differences in reactive oxygen species (ROS) production within the phagosomes [46]. This increased survival and phagosomal damage lead to greater IL-1β production through activation of the inflammasome via the NLRP6 receptor in macrophages [47]. NLRP6 is activated by LTA (Lipoteichoic acid), generally produced by Gram-positive bacteria [48]. While NLRP6 is highly expressed in intestinal epithelial and goblet cells, where it is involved in intestinal microbial homeostasis, epithelial cell repair, and regulation of mucus production [49–52], NLRP6 also functions within macrophages, where it can limit commensal driven inflammation while also having both protective and detrimental roles, as has been seen in *Listeria monocytogenes* infections [48,53,54]. As our previous studies have examined activated macrophage responses solely to streptococci *in vitro*, here we begin to examine the importance of *P. gingivalis* co-incubation in altering NLRP6 responses to *S. gordonii* both *in vitro* and *in vivo*.

## Methods

### Ethics statement

Mouse experiments were approved by the University at Buffalo Institutional Animal Care and Use Committee (IACUC) (protocol ID: PROTO202100042).

### Cell culture

RAW264.7 macrophages (ATCC) were grown in RPMI medium (Lonza or Corning) supplemented with 10% fetal bovine serum (FBS) (Corning) and 2 mM L-glutamine (Corning) at 37°C in 5% CO_2_. Prior to bacterial killing assays macrophages were stimulated with 20 ng/ml recombinant mouse IFN-γ (GenScript) for 24 hours and with 0.1 µg/ml lipopolysaccharide (LPS) (Salmonella enterica serotype Minnesota strain Re595; MilliporeSigma) for 2 hours to convert to activated (M1-like) macrophages.

Human monocytic THP-1 cells (ATCC) were maintained in RMPI medium supplemented with 10% FBS, 2 mM L-glutamine, 1 mM sodium pyruvate, 10 mM HEPES (Fisher Scientific), 1.5 g/L sodium bicarbonate and 0.05 mM 2-mercaptoethanol. Two days prior to an experiment, cells were differentiated to macrophages with 100 nM Phorbol 12-myristate 13-acetate (PMA) (Cayman Chemicals) for 24 hours then allowed to rest in media without PMA for an additional 24 hours [55]. Cells were stimulated with 20 ng/ml human IFN-γ for 24 hours then with 0.1 µg/ml LPS for 2 hours to activate towards an M1-like macrophage or left unstimulated [56]. For studies looking at cytokine production, cells were left unstimulated prior to the addition of bacteria.

Wild type (C57BL/6J) immortalized mouse bone marrow derived macrophages (iBMDMs) (provided by the lab of Dr. Gabriel Núñez, University of Michigan Medical School) were grown in RMPI medium supplemented with 10% FBS, 2 mM L-glutamine, 1 mM sodium pyruvate (Fisher Scientific). Cells were stimulated with 20 ng/mL mouse IFN-γ (GenScript) for 24 hours before experiments to differentiate to an M1-like macrophage or left unstimulated (M0 macrophages) [57].

### Microbial culture

*S. gordonii* strain DL1 (provided by Dr. Stefan Ruhl, University at Buffalo) were grown in brain heart infusion (BHI) medium (BD Biosciences) supplemented with 0.5% yeast extract (MP Biomedicals) at 37°C and 5% CO_2_. *P. gingivalis* (ATCC 33277 or W50) were grown anaerobically at 37°C in tryptic soy broth supplemented with menadione (1 µg/ml) and hemin (10 µg/ml). All experiments used mid-log phase bacteria cultures and MOI was calculated by counting using Petroff-Hausser Counter.

### Bacterial killing assay

To determine bacterial survival within macrophages a modified gentamycin resistance assay was used [46,58,59]. Briefly, macrophages were seeded in duplicate on 12-well plates. For activation (IFN-γ/LPS), macrophages were stimulated overnight with 20 ng/ml IFN-γ (human (BioLegend) or mouse (GenScript) as required), then with 0.1 µg/ml LPS for 2 hours. For experiments with dual incubation of *S. gordonii* and *P. gingivalis*, macrophages were not activated with IFN-γ or LPS. Mid-log growth *S. gordonii* was sonicated to break up chains and added to macrophages at a multiplicity of infection (MOI) of 10:1. Plates were centrifuged to synchronize contact of bacteria with macrophages (125 x g for 1 min). Cells were incubated at 37°C for 30 min, then one set of wells was washed extensively with PBS to remove external bacteria, macrophages were lysed with sterile H_2_O and serial diluted and plated on BHI or Todd-Hewitt (TH) plates to determine the initial number of bacteria taken up by the macrophages. For the other set of wells, 150 µg/ml gentamycin was added and incubated for 30 min at 37°C, after which the media was replace with fresh RPMI and incubated for an additional 1.5 hours. Again, macrophages were lysed with sterile H_2_O and serial diluted and plated on bacterial media plates and incubated overnight. After incubation, the number of CFU taken up and CFU survival 2 hours post-phagocytosis were determined. The ratio of surviving (2.5 hours) bacteria to phagocytosed (0.5 hours) bacteria gave us percent survival of bacteria within macrophages.

### Cytokine analysis

To measure cytokine release from THP-1 macrophages, cells were seeded on 12 or 24-well plates at 5 × 10^5^ cells/ml and left unstimulated for 2 hours at 37°C. Bacteria were then added at an MOI = 10:1, plates were centrifuged to synchronize contact of bacteria with macrophages (125 x g for 1 min) then incubated at 37°C for 6, 12, or 24 hours. After incubation cell supernatants were collected and spun down to remove cell debris and bacteria. Levels of TNFα, IL-6 and IL-1β were measured by ELISA (R&D systems) according to the manufacturer’s instructions. Concentrations (pg/ml) were normalized to amount per 1x10^5^ macrophages.

### Flow cytometry

Adherent cells were lifted by incubation with 0.5 mM EDTA at room temperature for 10 minutes and transferred to a 96-well v-bottom plate. All centrifugation was done at 4°C, 1500 rpm, for 8 minutes. Cells were blocked with mouse or human Fc Block (BD Biosciences) for 10 minutes at 4°C in 1% BSA in PBS. Cells were subsequently stained at 4°C and washed in BSA. For internal staining cells were fixed with 4% PFA for 20 minutes, then permeabilized with BD Perm/Wash buffer (BD Biosciences) according to manufacturer’s directions before staining with internal antibody. Flow cytometry was performed using a BD Fortessa flow cytometer, and all data were analyzed using FlowJo version 10.1 or higher.

### Ligature model of periodontal disease

Wild type control C57BL/6J (Jackson labs strain #000664) and NLRP6 knockout (*Nlrp6*^*-/-*^) mice (generously provided by Gabriel Núñez, University at Michigan [48]), 6–8 weeks in age, were divided into 5 groups (8–10 mice per group (4–5 female and 4–5 male)) as i) sham, ii) ligature alone, iii) ligature + *P. gingivalis,* iv) ligature + *S. gordonii*, and v) ligature + *P. gingivalis* & *S. gordonii*. After treatment with antibiotics to reduce, but not eliminate, the existing oral microflora (Kanamycin Sulphate 1 mg/ml for 5 days followed by a 3-day antibiotic free period), mice were intraperitoneally anesthetized with a 200 µl mixture of ketamine (10 mg/ml) and xylazine. A black braided silk ligature (6.0, Fisher Scientific) was placed on the left side second maxillary molars inoculated with 100 µl (1x10^9^ CFU/ml) of *S. gordonii* or *P. gingivalis,* or both, in 2% carboxymethyl cellulose (CMC), while the sham group received 2% CMC alone (Day-0). Booster doses with 100 µl of *P. gingivalis*, *S. gordonii* or both (1x10^9^ CFU/ml) were administered on the following Day-1 and 2 to the respective groups. After 14 days of ligature placement mice were sacrificed and the maxillary jaw bones were scanned with microCT. Briefly, the entire mouse head was harvested and fixed in 4% formaldehyde for high-energy microCT (Scanco100 μCT). Images were reconstructed and analyzed using Analyze Pro software (Analyze Direct, Inc. KS USA) to calculate the distance (micrometers) between the cemental epithelial junction (CEJ) to the alveolar bone crest (ABC) on the secondary molar at M-P (meso-palatal), D-P (disto-palatal), M-B (meso-buccal), D-P (disto-buccal) sites. The total alveolar bone loss was calculated by adding the distances at meso and distal points on both palatal and buccal sides.

Bone volume calculations were performed on the microCT image stacks using FIJI (ImageJ) [60]. Briefly, a reference-based registration [61] was applied to ensure consistent orientation and region-matched quantification across samples. A sham-control image was selected and manually oriented as the reference. A binary registration mask was created on the reference to outline the alveolar region surrounding the molars. Each sample was first manually aligned with the reference, then automatically refined using the registration mask, resulting in images with teeth and surrounding bone aligned. For quantification, a separate binary analysis mask was created on the reference image to define a standardized region of interest (ROI) around the second molar. The second molar was segmented and excluded before measurements. After registration, a FIJI script, available on GitHub, was used to batch-apply the analysis mask to all samples and extract bone/tissue density and bone volume fraction (BV/TV) from the same region in each microCT image.

### Bulk RNA preparation

RNA was extracted from collected tissue with Qiagen RNeasy kits. The extracted RNA was treated with DNAse1 using the New England Biolabs Monarch Spin RNA Cleanup Kit (T2020L). We used 100 ng of the total RNA as input for the Illumina Total Stranded RNA library prep kit with Ribo-Zero following the manufacturer's protocol. Per-cycle basecall (BCL) files generated by the NovaSeq 6000 were converted to per-read FASTQ files using bcl2fastq version 2.20.0.422 using default parameters (Illumina). The quality of the sequencing was reviewed using FastQC version 0.11.9 (Babraham Bioinformatics). Detection of potential contamination was done using FastQ Screen version 0.14 [62]. FastQC and FastQ Screen quality reports were summarized using MultiQC version 1.14 [63].

No adapter trimming was performed. Genomic alignments were performed using HISAT2 version 2.2.1 using default parameters [64]. Ensembl reference GRCm38 was used for the reference genome and gene annotation set. Sequence alignments were compressed and sorted into binary alignment map (BAM) files using samtools version 1.6.1. Counting of mapped reads for genomic features was performed using Subread featureCounts version 2.0.4 [65] using the parameters -p -s 2 –g gene_name –t exon –Q 60 -B -C, the annotation file specified with –a was the Ensembl GRCm38 reference provided by Illumina’s iGenomes. Alignment statistics and feature assignment statistics were again summarized using MultiQC.

### Bulk RNA sequencing analysis

Samples were analyzed using R (V4.5.0) with DESeq2 package (1.48.2) [66]. Basic quality control was performed by calculating total library size, the number of detected genes, and DESeq2 size factors for each sample. Sample counts were normalized using regularized log “rlog” and the within-group variability was calculated to perform differential expression analysis within each genotype. Differential expressions were determined by log fold changes, which were shrinkage-estimated using the apeglm method to improve the stability of effect size estimates [67]. Genes were classified as significantly up- or downregulated based on an adjusted p-value threshold and a minimum fold-change threshold. Volcano plots were used to visualize the upregulated and downregulated genes using ggplot2 (v3.5.2) package. Gene Ontology (GO) of biological processes and KEGG enrichment analysis were performed between WT and *Nlrp6*^*-/-*^ samples. To focus on immune-related biology, GO results were additionally filtered to the “immune system process” term. All enrichment plots were presented as bar charts using clusterProfiler (v4.16.0), with data obtained from the biomaRt database [68]. We set significance thresholds for all analyses, and significantly enriched terms were defined by an alpha value (Benjamini-Hochberg correction) of less than 0.05.

### Tissue staining

Sections from ﬁxed samples were stained with H&E using Hematoxylin (Gill III) (Sigma Aldrich) and Eosin-Y (Epredia), mounted using an immunohistochemistry mounting medium (Cell Signaling) and imaged on a Zeiss Axioskop. For Immunofluorescence staining, paraffin fixed decalcified slides were deparaffinized, then incubated in 0.2 M boric acid (Fisher Scientific) overnight at 60°C for antigen retrieval [69]. Primary antibody IL-1β (clone 3A6, Cell Signaling), neutrophil elastase (clone E8U3X, Cell Signaling) or F4/80 (clone T45-2342, BD Pharmingen) were used at a dilution of 1:200 with 0.5% Bovine Serum Albumin (Fisher) overnight at 4°C in a moisturized chamber. Secondary antibody (Alexa-488 or Alexa-594 conjugated donkey anti-mouse, Alexa-488 donkey anti-rabbit or Alexa-647 donkey anti-rat (Jackson ImmunoResearch)) were added in a dilution of 1:500 in 0.5% Bovine Serum Albumin (Fisher) for 1 hour at room temperature. Slides were counterstained with DAPI (Vector Laboratories) and mounted with 50% glycerol mounting media containing N-propyl gallate (MP Biomedicals). Images were taken on an Andor Dragonfly Confocal Microscope at the Optical Imaging and Analysis Facility (School of Dental Medicine, University at Buffalo). Image analysis was performed using FIJI [60].

## Results

### *P. gingivalis* macrophage stimulation enhances survival of *S. gordonii*

**Fig 1:**
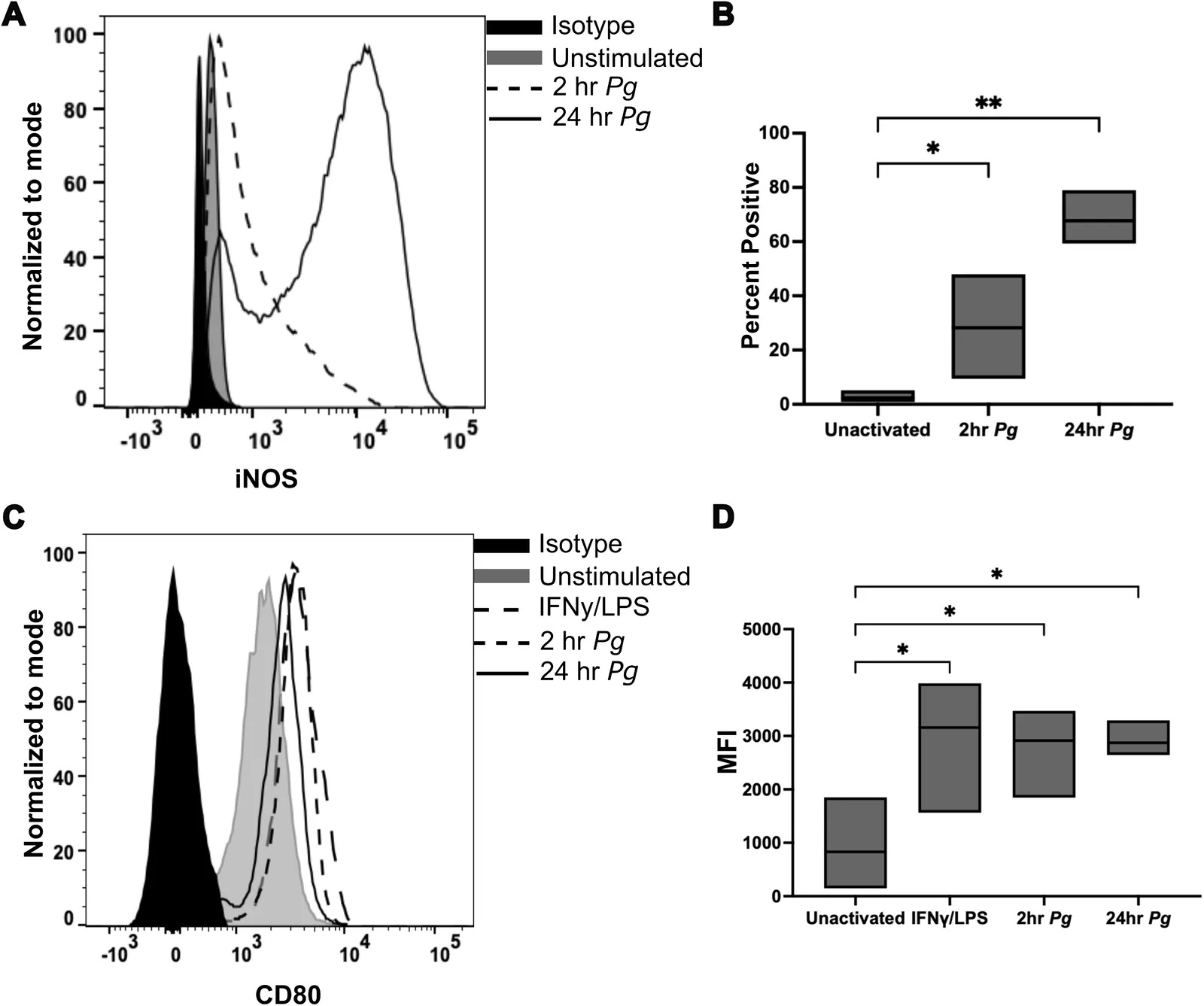
Inflammatory macrophage marker expression increases with *P. gingivalis* incubation. (A-B) Mouse bone marrow derived macrophages (BMDM) were differentiated with CSF1, then stimulated with *P. gingivalis* for 2 or 24 hours. Expression of iNOS was observed by flow cytometry. Quantitation (B) showed a significant increase after 2 and 24 hours, with an average of 28% positive after 2 hours and 68% after 24 hours. (C-D) PMA differentiated THP-1 macrophages were stimulated with *P. gingivalis* for 2 or 24 hours, or IFNγ/LPS for 24 hours as a positive control, and the mean fluorescence intensity (MFI) of CD80 was determined by flow cytometry. Quantitation showed a significant increase in MFI over control after 2- and 24-hours incubation with *P. gingivalis*. A and C are representative plots, B and D are min to max boxes with means (bars) of 3 independent experiments. P values (* < 0.05, ** < 0.01) were calculated by one-way ANOVA and Holm-Šídák’s multiple comparisons tests.

Direct interaction of *S. gordonii* and *P. gingivalis* can result in periodontal disease due to the ability of *S. gordonii* to facilitate the colonization and pathogenicity of *P. gingivalis* [2,21,70,71], though it is not clear if *P. gingivalis* can affect *S. gordonii* survival. Given our previous observations of increased *S. gordonii* survival and induction of IL-1β production in cytokine-induced inflammatory macrophages [46,47], we wanted to test if *P. gingivalis*-activated macrophages led to similar changes in *S. gordonii* survival. We first examined how *P. gingivalis* affects macrophage activation phenotypes. There was an increase in inflammatory macrophage (M1) markers CD80 and iNOS after stimulation with *P. gingivalis* for both 2 and 24 hours (Fig 1), agreeing with previous research showing *P. gingivalis* promotes an M1 phenotype while inhibiting an M2 phenotype [72–75]. This is similar to what has been found with cytokine activation of macrophages, which leads to prolonged reactive oxygen production in macrophage phagosomes [76] and allows for increased *S. gordonii* survival at multiple MOIs [46,47].

**Fig 2:**
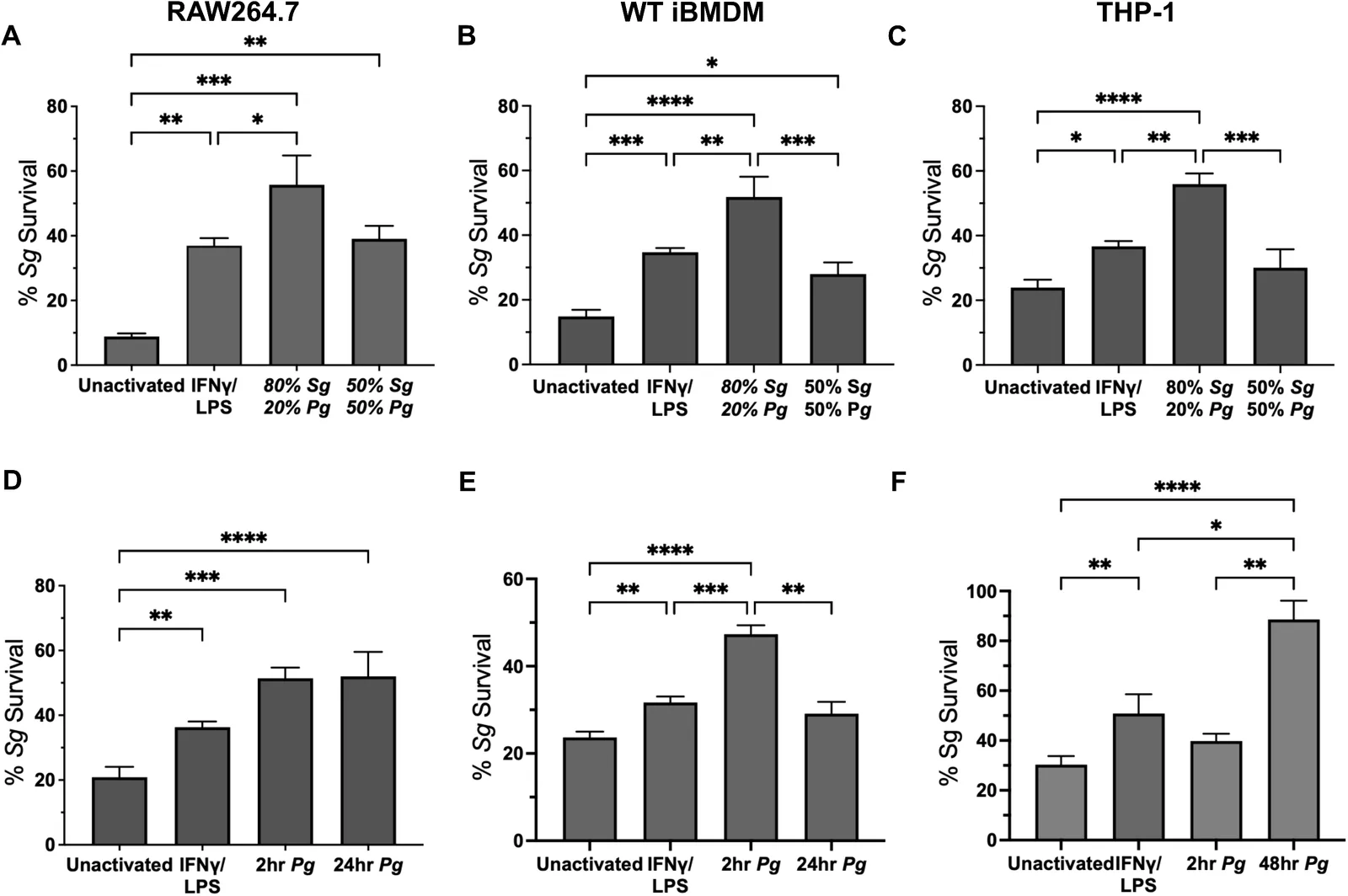
Survival of *S. gordonii* within macrophages increased with co-incubation of *P. gingivalis.* Shown is survival of *S. gordonii* within macrophages co-incubated with *P. gingivalis* at varying ratios (with a constant overall total MOI of 10:1) (A-C) or after stimulation with *P. gingivalis*, for the indicated times before incubation with *S. gordonii* (D-F); (A,D) RAW264.7 cells, (B,E) Immortalized mouse bone marrow derived macrophages (iBMDM) and (C,F) differentiated THP-1 cells. Shown are the means ± SEM of 3-8 independent experiments. P values (* < 0.05, ** < 0.01, *** < 0.001 and **** < 0.0001) were calculated by one-way ANOVA and Tukey’s multiple comparisons tests.

We next tested *S. gordonii's* ability to survive within macrophages when co-cultured with *P. gingivalis* (Fig 2). For these experiments, two different combinations of *S. gordonii* and *P. gingivalis* were used: 80% *S. gordonii* with 20% *P. gingivalis*, or 50% of each bacterium, while maintaining an overall MOI of 10:1 (see methods). With co-incubation of *S. gordonii* and *P. gingivalis*, *S. gordonii* survival in both mouse and human macrophages increased after phagocytosis. Interestingly, at the closer-to-physiologically relevant ratio where *S. gordonii* outnumbers *P. gingivalis*, we saw the highest percent internal survival of *S. gordonii* (Fig 2A–C).

We also tested *S. gordonii* survival in *P. gingivalis*-activated macrophages. Here we found that macrophages activated by *P. gingivalis* prior to the incubation of *S. gordonii* allowed for increased *S. gordonii* survival (Fig 2D–F), likely because *P. gingivalis* further stimulates macrophages to an inflammatory phenotype ((Fig 1) and [46,47]).

### Inflammatory cytokine release increased upon co-incubation with *S. gordonii* and *P. gingivalis*

**Fig 3:**
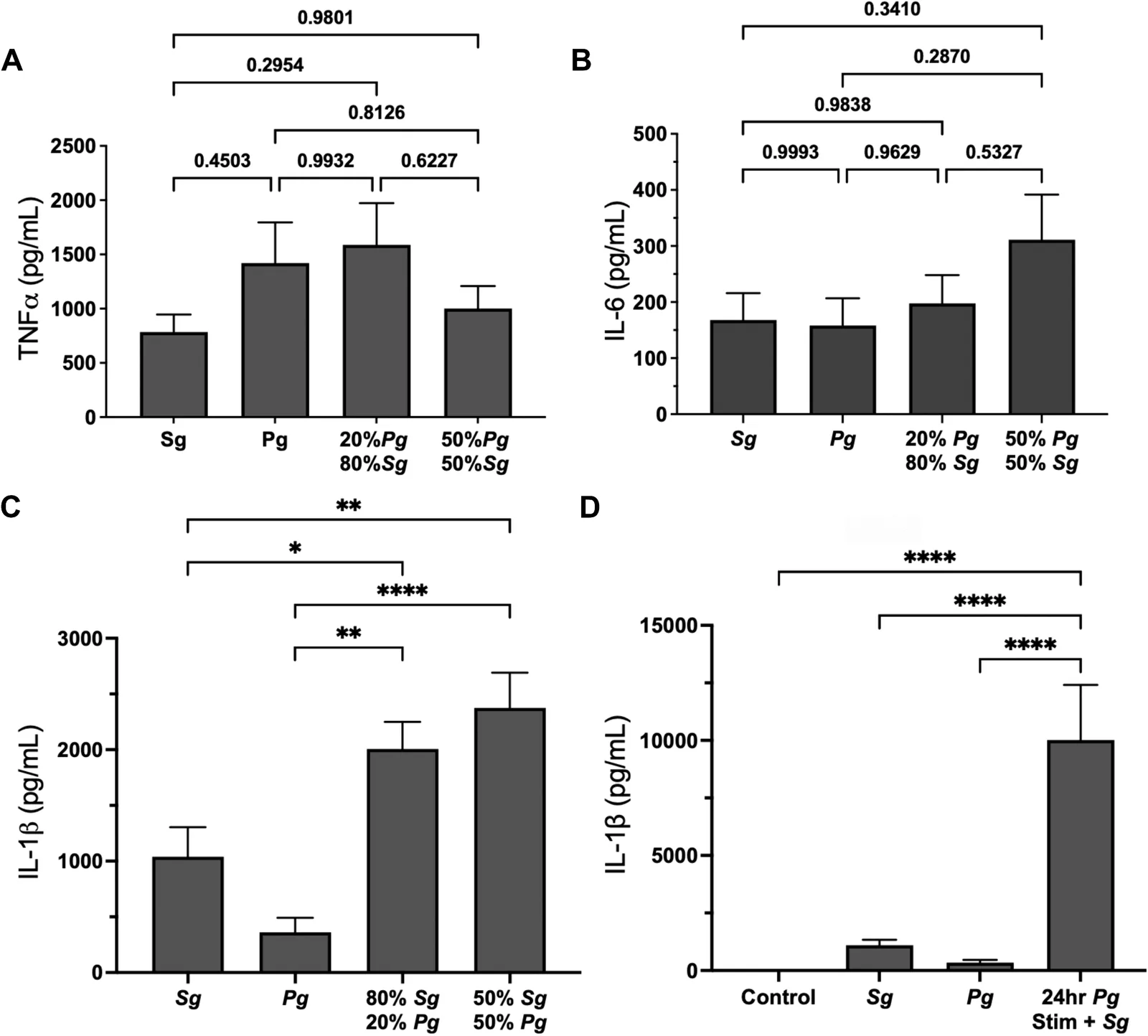
IL-1β, but not TNFα or IL-6, are increased following dual microbial incubation with macrophages. Inflammatory cytokine release from PMA differentiated THP-1 cells incubated with *S. gordonii, P. gingivalis,* or a combination thereof (All MOI = 10:1). (A) TNFα, (B) IL-6 and (C) IL-1β release after 6 hours incubation. (D) IL-1β release from THP-1 cells incubated with *S. gordonii* or *P. gingivalis* for 24 hours, or stimulated with *P. gingivalis* for 24 hours before incubating with *S. gordonii* for an additional 6 hours (24hr *Pg* Stim + *Sg*)*.* Shown are means ± SEM of 4 or more independent experiments. P values (in A-B, or * < 0.05, ** < 0.01, *** < 0.001 and **** < 0.0001 in C-D) were calculated by ordinary one-way ANOVA followed by Holm-Šídák’s multiple comparisons tests. No significant differences were seen with TNFα or IL-6 production.

Activated macrophages release a myriad of pro-inflammatory cytokines to elicit an immune response and recruit other immune cells. We examined a panel of three pro-inflammatory cytokines prevalent in periodontal disease, IL-1β, TNFα, and IL-6 [33,34,77,78] by human macrophages upon incubation with various combinations of *S. gordonii* and *P. gingivalis.* There were no significant changes in release of TNFα or IL-6 with co-incubation compared to incubation with *S. gordonii* or *P. gingivalis* alone (Fig 3A–B). There was, however, a significant increase in IL-1β production when *S. gordonii* was added simultaneously with *P. gingivalis* as compared to either bacterium alone (Fig 3C). An increase in IL-1β release was also seen when *P. gingivalis* strain W50, a generally more pathogenic strain in lesions [79,80] was used (S1 Fig). More significantly, when macrophages were stimulated with *P. gingivalis* for 24 hours prior to adding *S. gordonii,* there was a strong increase in IL-1β production (Fig 3D).

### Co-incubation of *P. gingivalis* and *S. gordonii* in animal model of periodontitis

We have previously reported that *S. gordonii* survival is increased within inflammatory macrophages [46], whereupon it can damage the phagosome and stimulate the inflammasome for IL-1β release via the NLRP6 pathway [47]. We found here that *P. gingivalis* can promote macrophages to an inflammatory phenotype (Fig 1), which is beneficial for *S. gordonii* survival and *S. gordonii*-induced IL-1β production [46,47]. We therefore next sought to determine whether disease development *in vivo* is dependent on NLRP6, specifically when both *P. gingivalis* and *S. gordonii* are present. Ligatures were placed on the second molar of either wild type (C57BL/6J) or *Nlrp6*^*-/-*^ mice and inoculated with a combination of *P. gingivalis* and *S. gordonii,* or each bacterium alone (see methods).

**Fig 4:**
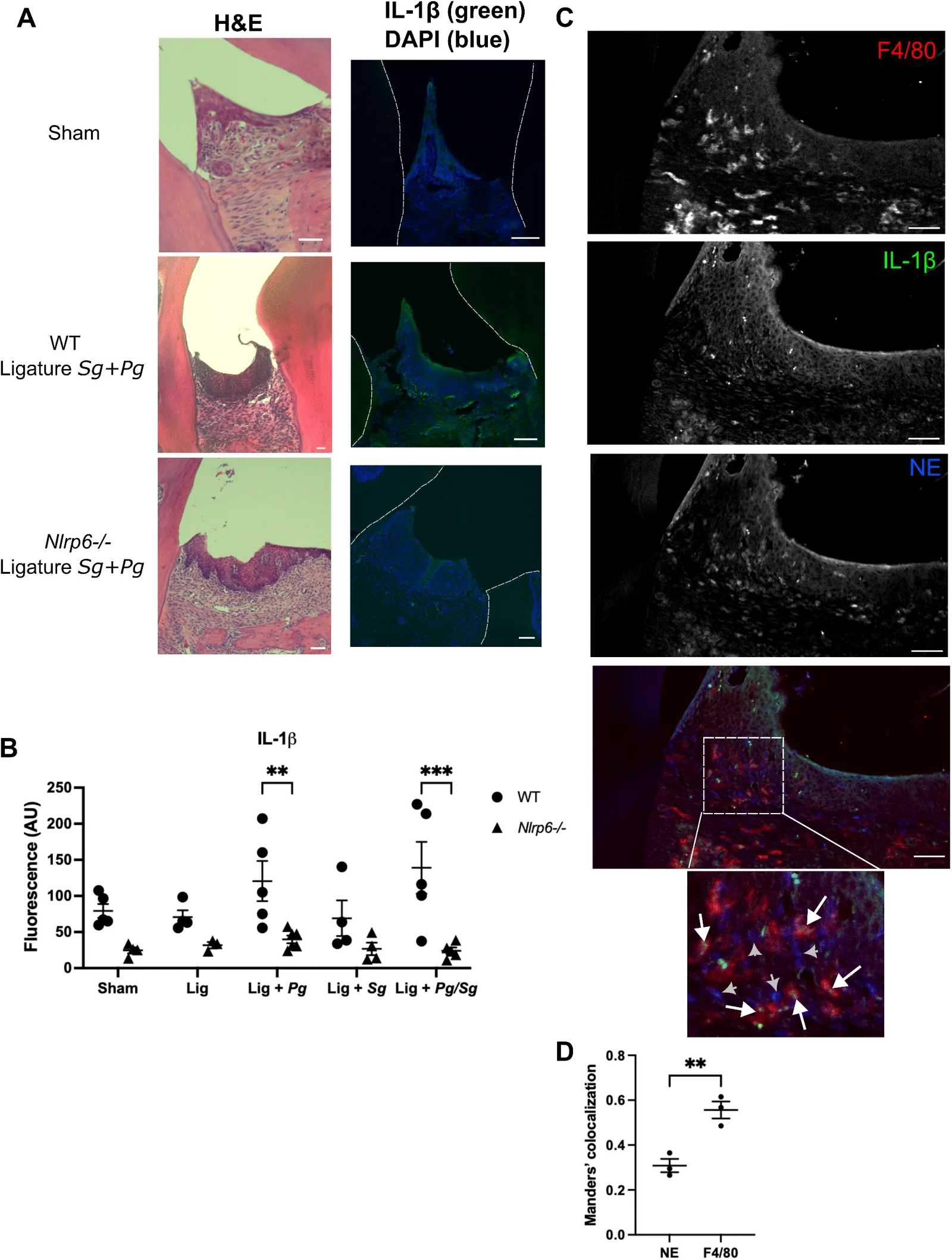
IL-1β production greatly reduced in gingiva of *Nlrp6*^*-/-*^ mice with ligatures and *P. gingivalis.* (A) Representative images of H&E-stained sections and immunofluorescence of IL-1β (green) and nuclei (DAPI, blue). Bars = 100 µm. (B) Quantitation of IL-1β immunofluorescence within the gingiva. Shown are individual mice data with means ± SEM indicated. P values (* < 0.05, ** < 0.01 and *** < 0.001) calculated by ordinary two-way ANOVA followed by Holm-Šídák’s multiple comparisons test, with a single pooled variance. (C) Representative image of WT mice with ligatures showing immunofluorescence of F4/80 (red) to label monocytes/macrophages, neutrophil elastase (NE, blue) to label neutrophils and IL-1β (green). Inset shows F4/80 co-localizing with IL-1β (white arrows) while neutrophil elastase shows less overlap (gray arrowheads). Bars = 50 µm. (D) Manders’ colocalization of IL-1β immunofluorescence with neutrophils (NE) or macrophages (F4/80) confirms higher IL-1β colocalization with macrophages. P value (** < 0.01) was calculated by ordinary unpaired t test.

Following tissue collection, H&E staining and immunohistology for IL-1β were performed on gingival tissue sections from the mice (Fig 4A). Quantification of IL-1β fluorescence showed significantly reduced IL-1β in *Nlrp6*^*-/-*^ mice that had ligatures with *P. gingivalis* as well as those that had ligatures with both *P. gingivalis* and *S. gordonii* (Fig 4B). Simultaneous staining of IL-1β, F4/80 (to label monocyte/macrophages), and neutrophil elastase (to label neutrophils) of WT mice showed much of the IL-1β detected was within the F4/80 labelled macrophages (Fig 4C–D), confirming earlier reports [81,82].

**Fig 5:**
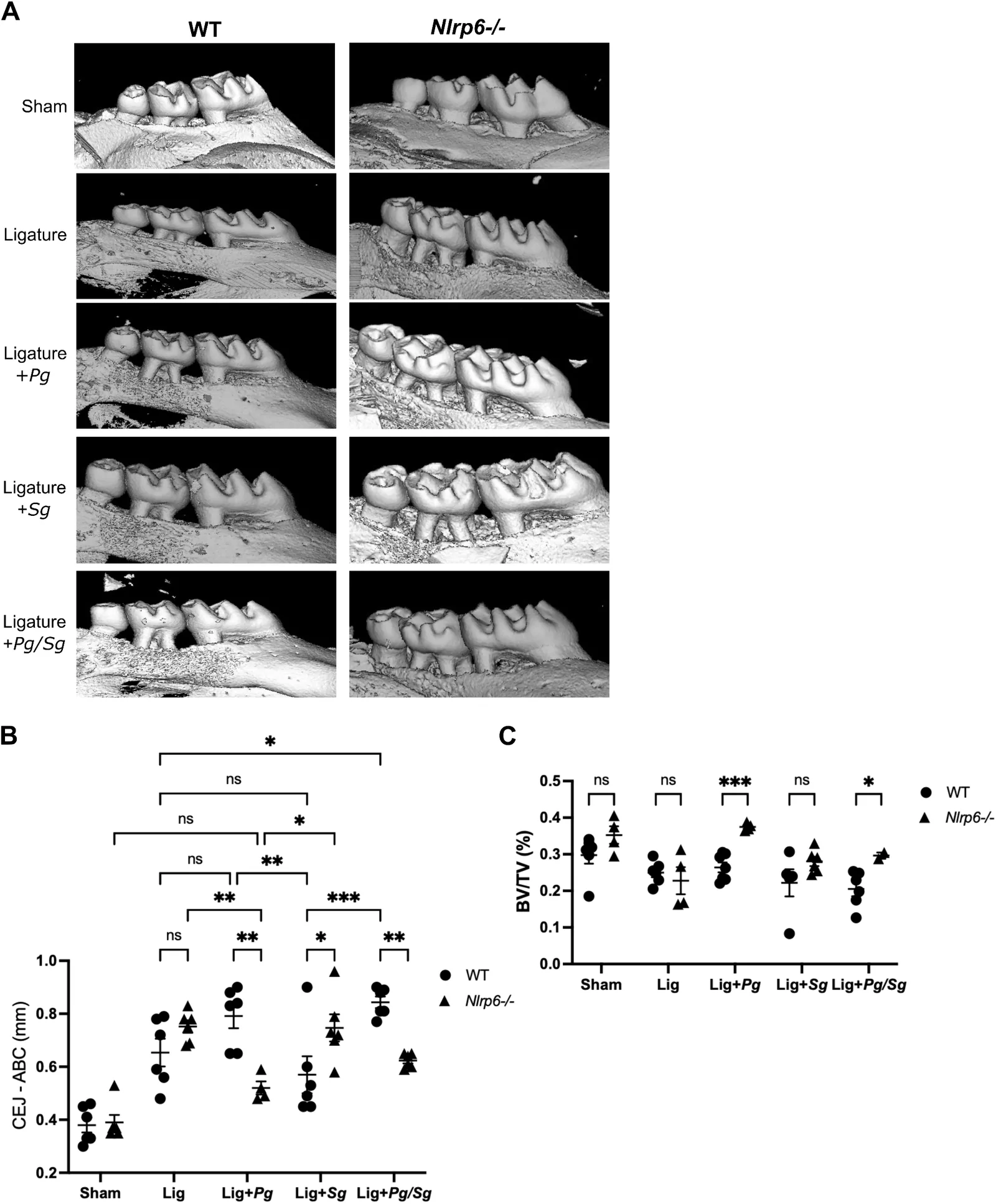
Bone loss in ligature-induced periodontal disease is significantly reduced in *Nlrp6*^*-/-*^ mice. (A) Sample microCT images of WT and *Nlrp6*^*-/-*^ maxilla after ligature (Lig) induced periodontitis. (B) Quantitation of cemental epithelial junction to alveolar bone crest (CEJ – ABC) distance measurements. Shown are means ± SEM, with P values (* < 0.05, ** < 0.01 and *** < 0.001; ns = not significant) calculated by ordinary two-way ANOVA followed by Holm-Šídák’s multiple comparisons test, with a single pooled variance. All mice with ligatures placed had significantly increased CEJ – ABC distances when compared to sham controls; for simplicity, only those not significant with sham are shown. (C) Quantitation of bone volume to total volume (BV/TV) fraction of microCT images. Shown are means ± SEM, with P values (* < 0.05 and *** < 0.001; ns = not significant) calculated by unpaired t test with Welch correction on each pair and Holm-Šídák’s multiple comparisons test.

MicroCT was also performed on the jaws to analyze bone loss (Fig 5). While all WT mice showed increased bone loss as measured by CEJ-ABC distance when a ligature was placed, mice that received both *P. gingivalis* and *S. gordonii,* but not *P. gingivalis* alone or *S. gordonii* alone, on the ligature had significantly increased CEJ-ABC distance than those with either ligature only (Fig 5B). *Nlrp6*^*-/-*^ mice that had ligatures with *P. gingivalis,* or both *P. gingivalis* and *S. gordonii*, had significantly reduced CEJ-ABC distance than the equivalent wildtype mice (Fig 5B). Intriguingly, *Nlrp6*^*-/-*^ mice with ligature alone or with *S. gordonii* only did not have decreased CEJ-ABC distance when compared to WT mice with the same treatment (p = 0.9 and 0.07, respectively). Bone volume to tissue volume (BV/TV) analysis of the microCT images found that, similar to CEJ-ABC distance results, BV/TV ratios were significantly increased (indicating less bone loss) in *Nlrp6*^*-/-*^ mice with ligatures incubated with *P. gingivalis* or both *P. gingivalis* and *S. gordonii* as compared to equivalent wildtype mice (Fig 5C). There were no significant changes in BV/TV in *Nlrp6*^*-/-*^ mice in sham, ligature alone or ligature with *S. gordonii*, similar to the CEJ-ABC results.

**Fig 6:**
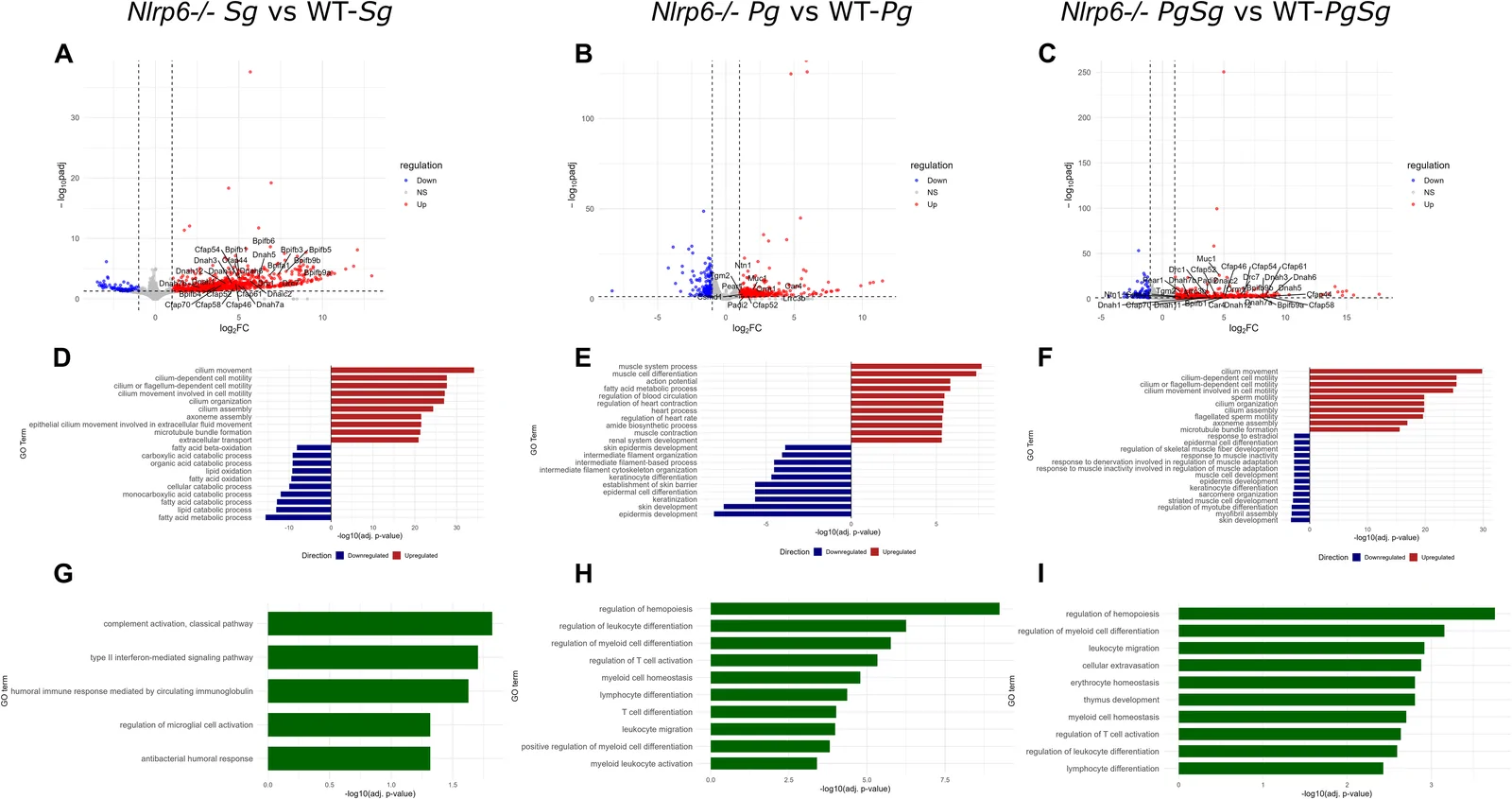
*Nlrp6*^*-/-*^ mice alter immune responses during periodontal disease. (A-C) Volcano plots showing differential gene expression between *Nlrp6*^*-/-*^ and WT treatments. Genes in blue are significantly downregulated; genes in red are significantly upregulated; genes in gray are not significantly changed between samples. (D-F) Gene Ontology (GO) enrichment analysis of biological processes was performed on *Nlrp6*^*-/-*^ mice compared with WT mice. (G-I) GO enrichment pathways focused on the immune system process term. For all GO analyses, significantly enriched terms were defined by an adjusted P value (Benjamini-Hochberg correction) of less than 0.05.

We also examined bulk mRNA isolated from the gingival tissue of the mice (Fig 6). There were no significant differences in inflammatory cytokine production associated with periodontal disease (TNFα, IL-6, IL-17), with the exception of IL-1β which was reduced in *Nlrp6*^*-/-*^ mice. There were also no significant changes in other inflammasome components, including ASC (pycard) or NLRP3. In general, all *Nlrp6*^*-/-*^ samples exhibited more upregulated genes compared to their WT counterparts. Both *Nlrp6*^*-/-*^ groups with *S. gordonii* (*Sg* and *Sg/Pg*) showed increased expression of genes related to cell motility, such as the Dnah family (Dnah1, Dnah3, Dnah5, Dnah6, Dnah7a, Dnah7b, Dnah11, Dnah12, Dnaic2, Drc1, Drc7) and the Cfap family (Cfap44, Cfap46, Cfap52, Cfap54, Cfap58, Cfap61, Cfap70), as well as genes involved in antimicrobial defense from the Bpifb family (Bpifb1, Bpifb9a, Bpifb9b) (Fig 6A,C). We conducted a GO enrichment analysis on the gene sets and the top pathways upregulated in the *Nlrp6*^*-/-*^ *S. gordonii* samples were consistent with what was observed in the volcano plots, related to cell motility (Fig 6A,C). In contrast, the WT samples showed upregulation of pathways involved in fatty acid metabolism. When the GO analysis was further refined to focus on immune-related pathways, the most enriched pathways in *S. gordonii*-inoculated samples involved the classical inflammatory response (complement activation) and the alternative immune response (type II interferon, humoral immune response, and antibacterial humoral response) (Fig 6A,C).

The addition of *P. gingivalis* (*Pg* and *Sg/Pg*) induced inflammation, as seen with increased bone loss. Bulk-RNA analysis revealed that both *Nlrp6*^*-/-*^ groups with *P. gingivalis* upregulated multiple genes involved in inflammation regulation (Csmd1, Padi2, Tgm2, and Orm1) and epithelial immune functions (Muc1, Pear1, Ntn1, Lrrc3b, and Car4) (Fig 6B,C). GO enrichment analysis revealed the top pathways upregulated in *P. gingivalis Nlrp6*^*-/-*^ samples were related to metabolic changes and fatty acid metabolism (Fig 6B,C). WT samples had upregulated pathways related to epithelial function (Keratinocyte differentiation, establishment of skin barrier, epidermis development). *Pg-Sg Nlrp6*^*-/-*^ samples upregulated pathways similar to *S. gordonii Nlrp6*^*-/-*^ related to cilium movement, while the *Pg-Sg* WT samples had similar pathways upregulated to *P. gingivalis* WT samples in epithelial function (Fig 6B,C). The refined immunological GO pathway analysis revealed *P. gingivalis* samples showed an enrichment of pathways in the regulation of myeloid cell differentiation, leukocyte migration, and T cell signaling (Fig 6B,C).

Overall, the RNAseq data suggests that during *P. gingivalis* inflammation, in the *Nlrp6*^*-/-*^ samples, the epithelial cells play a more crucial role, whereas in the WT sample, possibly in part due to the excessive inflammation induced by IL-1β, epithelial cells are attempting to restore the barrier. Additionally, although *S. gordonii* alone does not induce excessive inflammation and IL-1β production, *Nlrp6*^*-/-*^ mice showed altered immune signaling, metabolism, and cellular motility in response to excessive Gram-positive bacteria.

**Fig 7:**
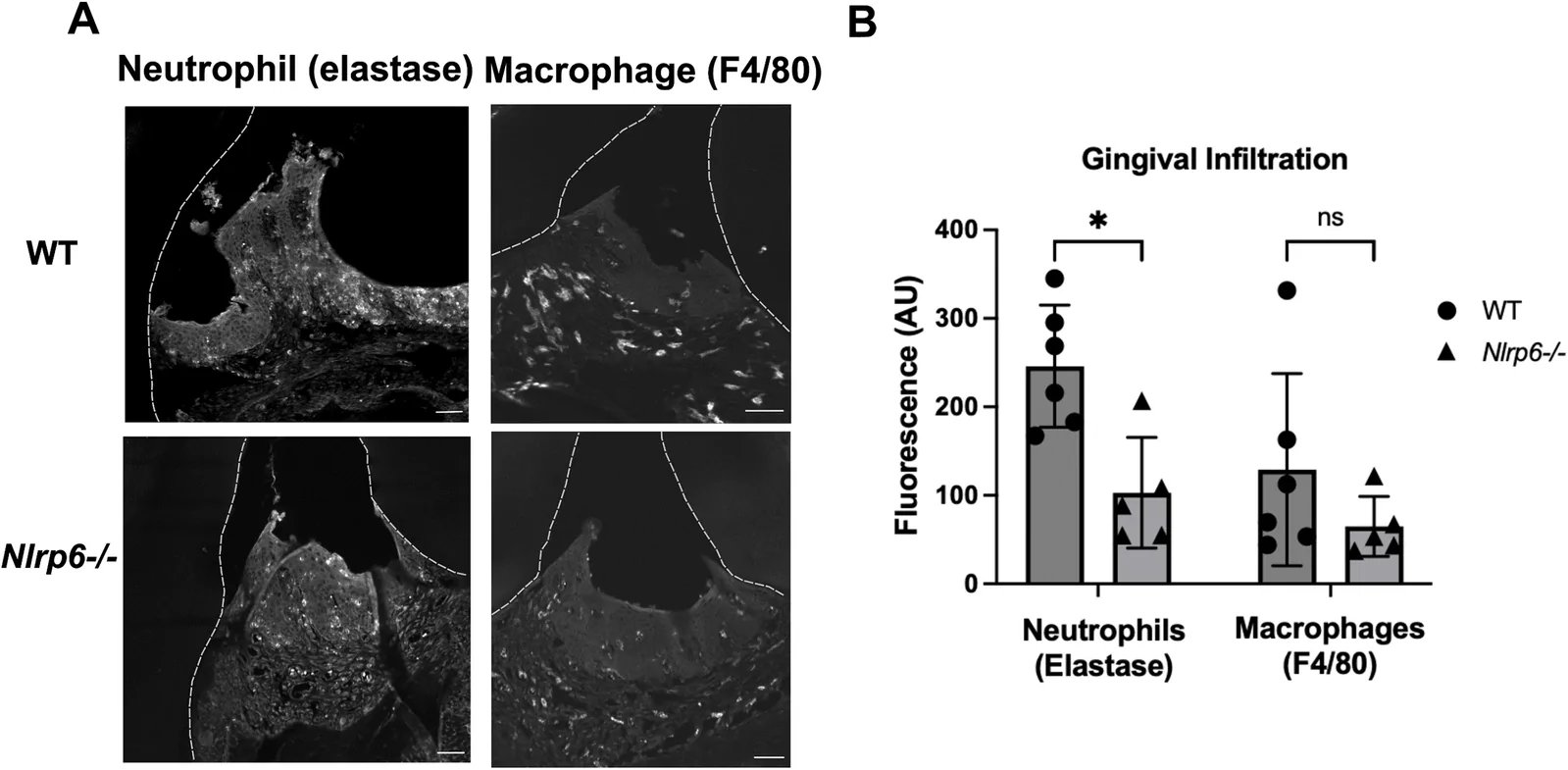
Gingiva neutrophil infiltration is reduced in *Nlrp6*^*-/-*^ mice. (A) Representative images of mice with ligatures plus *S. gordonii* and *P. gingivalis* showing immunofluorescence of F4/80 to label monocytes/macrophages and neutrophil elastase to label neutrophils. Bars = 50 µm (B) Quantitation of F4/80 and neutrophil elastase immunofluorescence within the gingiva. Shown are means ± SEM, P values (* < 0.05, ns = not significant) were calculated by t tests with Welch correction and Holm-Šídák’s multiple comparisons test.

Because we observed changes in cell motility pathways, we examined the levels of innate immune cell infiltration (neutrophils and monocytes/macrophages) using immunofluorescence of tissue sections with antibodies to neutrophil elastase and the macrophage marker F4/80 (Fig 7). Quantitation revealed a significant decrease in neutrophils within the marginal gingival tissue in *Nlrp6*^*-/-*^ mice when a ligature was present as compared to WT mice (Fig 7B).

## Discussion

IL-1β is well-known to be involved in the pathogenesis of chronic inflammatory diseases, including periodontal disease [41–44]. While there are many activators of the inflammasome, the complex that activates caspases responsible for the maturation and release of IL-1β [83,84], to date most studies investigating IL-1β and inflammasome involvement in periodontal disease have focused on the role of the activator NLRP3 [85–92]. Our results reveal that the inflammasome activator NLRP6 is an important player in periodontal disease development.

In the oral cavity Gram-positive bacteria, which are the initial colonizers of the tooth surface, outnumber late colonizing Gram-negative anaerobes both in a healthy oral environment and during gingivitis development [93–95]. In addition, while generally reduced as a percent of total bacteria present, Gram-positive bacteria are still a significant community member during advanced periodontal disease [2,5,94–96]. Indeed, the numbers of all microbial members interacting with the immune system can be significant at all stages of health and disease [3,13,30], however, keystone pathogens such as *P. gingivalis* can dysregulate both the oral microbiome and the host immune response to promote disease [13,97]. Our initial *in vitro* results confirmed the ability of *P. gingivalis* to activate macrophages toward an M1 phenotype (Fig 1) and induce some IL-1β production (Fig 3). Similarly, *S. gordonii* incubation with macrophages alone, without pre-activation, was insufficient to elicit a robust IL-1β response (Fig 3 and [46]). We have also previously reported that changes in MOI of single bacterial incubations with macrophages, from 10 to 100, does not significantly change the levels of IL-1β release [98]. In contrast, co-incubation with *P. gingivalis* and *S. gordonii* at a combined MOI of 10:1 induced substantially greater IL-1β production than either species alone, adding to our previous work showing that oral streptococci increase IL-1β production via activation of the NLRP6 inflammasome when engulfed by activated phagocytes [47,99]. In addition to an increase in IL-1β production, we observed an increase in *S. gordonii* survival in macrophages activated by *P. gingivalis* in a variety of macrophage cell lines, including the RAW264.7 cell line, as we’ve seen previously [46,47]. Given RAW264.7 cells lack ASC [100], a crucial central adaptor molecule for the inflammasome pathway, these results suggest *S. gordonii* survival is not dependent on inflammasome activation by *P. gingivalis*. Here, we saw macrophage activation by *P. gingivalis*, together with *S. gordonii*-mediated NLRP6 signaling, induced robust IL-1β release above what either bacterium was able to induce independently. Given that *P. gingivalis* is known to activate AIM2 and NLRP3-dependent inflammasomes [101–105], the enhanced IL-1β response may be due to the activation of multiple inflammasome pathways. These results are consistent with previous studies demonstrating enhanced cytokine production following exposure to multiple oral bacterial species [106,107].

To understand the *in vivo* contribution of NLRP6 signaling to the inflammatory response, and periodontal disease development, induced by co-infection with *P. gingivalis* and *S. gordonii*, we used an NLRP6 knockout mouse model. In the presence of *P. gingivalis*, *Nlrp6*^*-/-*^ mice exhibited significantly reduced disease severity, as measured by reduced bone loss and IL-1β production in the ligature model. These findings provide further evidence that *P. gingivalis* relies on the normally commensal oral microbiome [2], including oral streptococci, not only for colonization [21,22], but also to promote dysbiosis. Our findings further demonstrated that the addition of *P. gingivalis* alone to ligatures did not significantly increase bone loss when compared to ligatures alone, though it did increase bone loss compared to ligatures with *S. gordonii* alone (Fig 5). The addition of ligatures can cause significant disease and bone loss due to increased microbial load [108–110], but without a robust microbiome to assist with its colonization, *P. gingivalis* cannot cause disease [2,21,111]. Most mouse studies examining *P. gingivalis*-induced periodontal disease use a gavage model (without ligatures) over a time course of ~6 weeks [2,21,89,112,113]. The results presented here suggest cooperation between *S. gordonii* and *P. gingivalis* can cause increased disease over ligature addition alone. Further, when combined with our previous results showing incubation with inflammatory-activated macrophages allows for increased *S. gordonii* survival within, and escape from, these macrophage phagosomes [46] and subsequent NLRP6 inflammasome activation [47], the results presented here suggest that the keystone bacterium *P. gingivalis* not only enhances inflammation in a positive feedback loop which further promotes dysbiotic microbial growth [13], but also allows *S. gordonii* to actively promote inflammation via NLRP6 inflammasome activation.

While periodontal bone loss was decreased in *Nlrp6*^*-/-*^ mice as compared to WT mice when *P. gingivalis* was added to the ligatures, either alone or with *S. gordonii, S. gordonii* alone in *Nlrp6*^*-/-*^ mice did not show bone loss changes when compared to WT mice. *S. gordonii* is part of the normal healthy microflora, and under these conditions interactions of the eubiotic microbiome with the immune system is generally homeostatic [14]. As we have previously shown, NLRP6-dependent, *S. gordonii*-mediated increases in IL-1β release from innate immune cells only occurs when the immune cells have been previously activated [46,47,99]. This suggests that detrimental effects of NLRP6 in the oral environment may become important only during persistent inflammation. This aligns with previous research on the role of NLRP6 in the gut, which found it to have a regulatory and protective role in many infections, but can be detrimental in cases of chronic inflammation [48,49,53,114,115]. The requirement for enhanced inflammation *in vivo* likely stems from the presence of *P. gingivalis*, which is known to influence the immune system [1,14,116] and, as we showed here, can influence IL-1β production by macrophages in response to S*. gordonii*. When *P. gingivalis* is added alone without *S. gordonii*, the mouse’s naturally present commensals can partially enhance the effect of *P. gingivalis* [2]. Indeed, we have seen that activated macrophages have altered responses to other oral streptococcal species [47], though a thorough investigation into the survival and IL-1β induction ability of additional oral streptococci species is not yet complete. Similarly, the ability of Gram-negative keystone oral pathogens, other than *P. gingivalis,* to activate immune cells and allow for increased survival of *S. gordonii* (which is predominantly in gingival plaque [117]), or other oral streptococci, leading to NLRP6-dependent IL-1β production is under active investigation.

Part of the difference in response with and without *P. gingivalis* added to the ligatures may also be due to a shift in the major cell type responding with NLRP6 signaling. Studies of gut infections, where NLRP6 is highly expressed in epithelial cells [118], suggest NLRP6 plays a mostly protective role [48,53], while NLRP6 appears to promote disease when myeloid cell NLRP6 responses dominate [49,115,119]. The relative expression of NLRP6 in different cells within the oral cavity is unclear, though it can activate the inflammasome and regulate cytokine responses in periodontal ligament cells and gingival fibroblasts [120,121]. Studies to understand NLRP6 within the oral cavity environment and its role in regulating the oral microbiome are therefore an important area for future research. Related, the interconnection between NLRP6 and other inflammasome activators implicated in periodontal disease, including NLRP3 and AIM2, that can be activated by *P. gingivalis* [87,90,101,104,105], and their relative importance in disease, is an area that needs clarification. Other than IL-1β, our results indicated other inflammatory cytokines often associated with periodontal disease, TNFα and IL-6, had no change in expression. This may partly explain why no changes were seen in *Nlrp6*^*-/-*^ mice with ligature alone or with ligature and *S. gordonii* alone; these cytokines also play a role in periodontal bone loss [30,82] and are likely important in the milder bone loss seen under these conditions.

Neutrophils are critical mediators of inflammation during periodontal disease development, and the number of neutrophils in *Nlrp6*^*-/-*^ mice was significantly reduced, though not eliminated. This is important as increased neutrophils correlate with disease models and clinical data [122,123], whereas neutropenia is also associated with poor oral health [124]. However, how reduced IL-1β production in *Nlrp6*^*-/-*^ mice, or the absence of NLRP6 itself, affects the adaptive immune cells involved in periodontal disease, such as Th17 cells, remains an open question. While IL-1β appears not to be important for the direct recruitment and expansion of Th17 cells, which are implicated in periodontal disease [125], we observed broad overall changes in T cell signaling in our RNAseq data. Parsing out how these changes specifically alter the adaptive immune landscape [30] will be important to understand in future studies. It will also be important to further understand the role of the NLRP6 inflammasome in periodontal disease-linked development of innate trained immunity and systemic diseases [126,127].

In summary, our results indicate that NLRP6 plays an important role in IL-1β production and periodontal disease progression in the ligature-induced model. Since NLRP6 is activated by Gram-positive LTA [48], its role may lie in enabling normally commensal members of the oral microbiome to become pathobionts when a keystone pathogen, or other initiator of disease or inflammation, is present.

## Supporting information

S1 Fig IL-1β release from macrophages increased with co-incubation of *P. gingivalis* strain W50. IL-1β release from PMA differentiated THP-1 cells incubated with *S. gordonii*, *P. gingivalis* strain W50, or a combination thereof (all total MOI = 10:1) after 6 hours incubation. Shown are means ± SEM of 3 independent experiments. P values (* < 0.05) were calculated by ordinary one-way ANOVA followed by Holm-Šídák’s multiple comparisons tests.
(PDF)
