# Supplementary material for: Polymicrobial-driven NLRP6 inflammasome regulates IL-1β production and alveolar bone loss in a murine model of periodontitis
Source: PLoS Pathog. 2026 Sep 15;22(9):e1014574. doi: 10.1371/journal.ppat.1014574 (PMC13588512; doi:10.1371/journal.ppat.1014574)
Supplement: Supplementary file 1 [file ppat.1014574.s001.pdf]

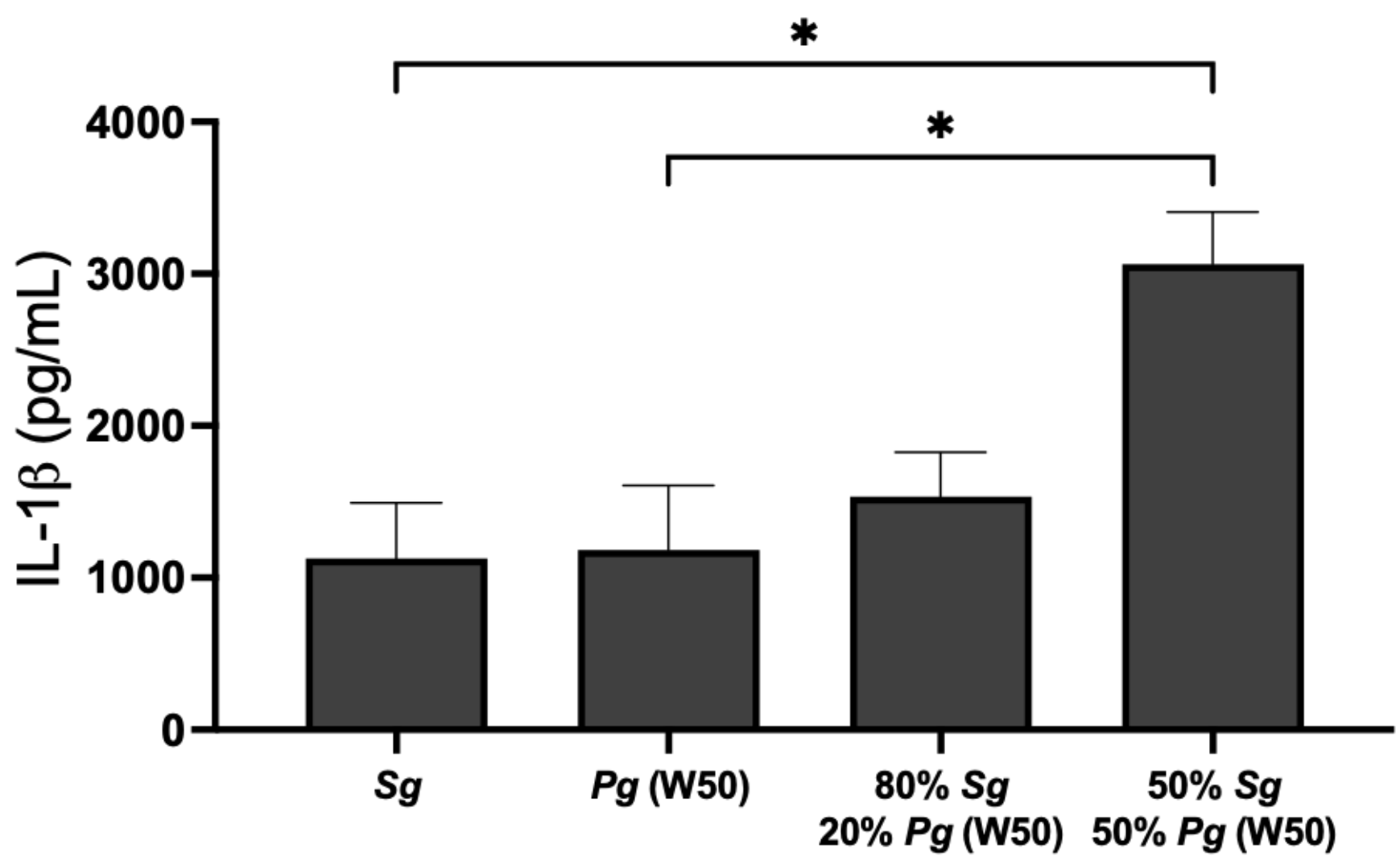

Figure S1. IL-1β release from macrophages increased with co-incubation of P. gingivalis strain W50. IL-1β release from PMA differentiated THP-1 cells incubated with S. gordonii, P. gingivalis strain W50, or a combination thereof (all total MOI = 10:1) after 6 hours incubation. Shown are means ±SEM of 3 independent experiments. P values (*<0.05) were calculated by ordinary one-way ANOVA followed by Holm-Šídák's multiple comparisons tests.
