# Supplementary material for: Polymicrobial-driven NLRP6 inflammasome regulates IL-1β production and alveolar bone loss in a murine model of periodontitis
Source: PLoS Pathog. 2026 Sep 15;22(9):e1014574. doi: 10.1371/journal.ppat.1014574 (PMC13588512; doi:10.1371/journal.ppat.1014574)
Supplement: Supplementary file 2 [file ppat.1014574.s003.pdf]

University at Buffalo
School of Dental Medicine

2026-07-29

Drs. Hakansson, Wessels and reviewers,

We are submitting a revised version of the manuscript "**Polymicrobial-driven NLRP6 inflammasome regulates IL-1β production and alveolar bone loss in a murine model of periodontitis**".

We thank the reviewers for their time and thoughtful comments for our initial submission. We have modified the manuscript in response to the comments, and our replies to specific inquiries are included below.

All authors have reviewed and approved the revised manuscript. For any concerns, please contact me as the corresponding author.

Regards,

Jason Kay, MSc, PhD
Associate Professor
Director of Oral Sciences MS program

Oral Biology
Microbiology and Immunology

545 Biomedical Research Building, Buffalo, NY 14214-8024
+1.716.829.2073
jasonkay@buffalo.edu

dental.buffalo.edu

Comments:

1. Mechanistic interpretation and causal relationships: While the data support an important role for NLRP6 in the observed phenotype, the proposed mechanistic pathway linking P. gingivalis-induced macrophage polarization, enhanced bacterial persistence, NLRP6 activation, and increased IL-1β production is not fully established by the current data. If additional experimental evidence supporting this mechanistic model is available, it would considerably strengthen the manuscript. Otherwise, I ask that you revise the text to more clearly distinguish experimentally demonstrated findings from mechanistic interpretations and explicitly discuss these limitations.
We have revised the discussion throughout to make it clearer as to what we have shown mechanistically here, what we have shown previously, and what remains to be formally tested.

2. Generalizability of the findings: The study relies on single strains of both P. gingivalis and S. gordonii. Although I do not consider this a major limitation of the study, the manuscript would benefit from a discussion of how strain-to-strain variability or the use of other periodontal pathogens might influence the broader applicability of the findings.
We have added a supplemental figure showing results obtained with a different strain of *P. gingivalis* (W50), and have modified the discussion (including specifically lines 470-473) to address this concern.

3. Physiological relevance of the experimental infection conditions: The infection model employs a multiplicity of infection that is commonly used. However, it would be helpful to discuss the physiological relevance of these conditions. If similar observations were obtained using lower bacterial inocula, please include this information. Otherwise, please address this point in the Discussion.
We have modified the text, including at lines 284, 402-406 and 410-416, in an attempt to address this concern, indicating we have seen *S. gordonii* survival and IL-1β production induction at lower MOIs (Croft et al 2018 and Metcalfe et al 2023, respectively) and that the physiological relevance of the MOIs used are relevant to disease development, where significant levels of oral microbial biofilms (plaque) exist and high levels correlate with disease.

4. Discussion and interpretation of the in vivo findings: I encourage you to further strengthen the Discussion by placing the findings into the broader context of polymicrobial host-pathogen interactions and innate immune regulation in periodontal disease. In addition, please ensure that the conclusions drawn from the ligature model are appropriately balanced, particularly if bacterial challenge does not significantly increase bone loss beyond that observed with ligature alone.
The discussion has been broadened (including spciefically at lines 435-450) to address these concerns, specifically discussing previous work detailing known interactions of *S. gordonii* and *P. gingivalis* and the fact that ligature alone will cause some disease and potential reasons for that.

Reviewer #1:
1. Data in Fig1 should include a statistical analysis
We performed statistical analysis and added the results to Figure 1.

2. Fig 2 D, E, F, provide some explanation for the different pattern with the RAW264.7 cells
We're not sure which pattern the reviewer is referring to, but there does appear to be a potential difference in survival of *S. gordonii* between RAWs and other macrophage lines. The RAW cell line is known to be missing the inflammasome central component ASC, so, while changes in phagosome ROS production will allow increased *S. gordonii* survival, the inflammasome, and more specifically pyroptosis, which can increase intracellular bacterial killing, is not activated in these cells. This may account for more survival of *S. gordonii* in RAW cells after 24 hours. However, we do not want to overanalyze these results so other than making a comment in the results about the lack of ASC in RAW cells (line 416-420), we have not further speculated.

3. Fig 3C. Is there a statistical difference compared to S. gordonii alone, this should be shown, as the text indicates that it is S. gordonii that induces IL-1β.
Statistics were added for *S. gordonii*, but no significant differences in IL-1β production between individual incubations. *P. gingivalis* can induce IL1 through NLRP3 and AIM2 (as discussed in manuscript), and significantly more IL-1β is produced when both bacteria are present than with either alone, and we showed this is dependent on NLRP6. We have updated the text in results (lines 305-307) and discussion (lines 421-427) to indicate more clearly that the combination of bacteria, not either alone, is required for maximum IL-1β production.

4. Fig 3 A-C. For completeness, experiments should be run with P. gingivalis and S. gordonii alone at numbers equivalent to those used in the mixtures
We have previously published data with varying MOIs and IL-1β production (Varanat 2017) and have found that even with a 10-fold change in MOI of single incubations with macrophages there are no significant changes in IL-1β production. This information has been added to the discussion (including in lines 410-412).

5. Discuss if the effect of P. gingivalis is specific to this organism or would other periodontal pathogens show the same phenotype
We imagine any Gram-negative periodontal pathogen that activates macrophages will allow for increased S. gordonii survival and will have a similar effect, and are indeed testing additional pathogens. The discussion has been updated (including specifically lines 470-473) to address this.

6. Fig 4C. A quantitative analysis of co-localization should be performed
This has been performed and added to Figure 4 (D).

7. Fig 5B. There appears to be no statistical difference in bone loss in WT between ligature alone and ligature + P. gingivalis. This indicates that bacteria are not the primary driver of bone loss in this model, so results should be interpreted more cautiously.
We have expanded the discussion to address this, including specifically in lines 330-334 and 432-444.

8. Line 325 and elsewhere. Please describe how the WT and ko mice were 'matched'
Removed word matched in these instances and indicated that the WT and knockout mice with identical treatment were what are being compared.
