# Supplementary material for: Polymicrobial-driven NLRP6 inflammasome regulates IL-1β production and alveolar bone loss in a murine model of periodontitis
Source: PLoS Pathog. 2026 Sep 15;22(9):e1014574. doi: 10.1371/journal.ppat.1014574 (PMC13588512; doi:10.1371/journal.ppat.1014574)
Supplement: Peer review history

# Peer review: Decision Letter 0

**DOI:** 10.1371/journal.ppat.1014574.r001

**Authors:** Michael Wessels, Anders Hakansson

2 Jul 2026

Polymicrobial-driven NLRP6 inflammasome regulates IL-1β production and alveolar bone loss in a murine model of periodontitis

PLOS Pathogens

Dear Dr. Kay,

Thank you for submitting your manuscript to PLOS Pathogens. After careful consideration, we feel that it has merit but does not fully meet PLOS Pathogens's publication criteria as it currently stands. Therefore, we invite you to submit a revised version of the manuscript that addresses the points raised during the review process.

Please submit your revised manuscript by Aug 31 2026 11:59PM. If you will need more time than this to complete your revisions, please reply to this message or contact the journal office at plospathogens@plos.org. When you're ready to submit your revision, log on to https://www.editorialmanager.com/ppathogens/ and select the 'Submissions Needing Revision' folder to locate your manuscript file.

Please include the following items when submitting your revised manuscript:

* A letter that responds to each point raised by the editor and reviewer(s). You should upload this letter as a separate file labeled 'Response to Reviewers'. This file does not need to include responses to any formatting updates and technical items listed in the 'Journal Requirements' section below.

* A marked-up copy of your manuscript that highlights changes made to the original version. You should upload this as a separate file labeled 'Revised Manuscript with Track Changes'.

* An unmarked version of your revised paper without tracked changes. You should upload this as a separate file labeled 'Manuscript'.

We look forward to receiving your revised manuscript.

Kind regards,

Anders P Hakansson, Ph.D.

Academic Editor

PLOS Pathogens

Michael Wessels

Section Editor

Editor-in-Chief

PLOS Pathogens

orcid.org/0000-0003-2946-9497

Michael Malim

Editor-in-Chief

PLOS Pathogens

orcid.org/0000-0002-7699-2064

**Additional Editor Comments (if provided):**

I apologize for the extended time it has taken to reach this decision. We experienced unusual difficulty in securing appropriate expert reviewers for your manuscript despite extensive efforts. Therefore, the present decision is based on the detailed external review that was obtained together with my own editorial assessment of the manuscript below.

The reviewer found the study to be well performed and considered the work to provide novel insights into the role of NLRP6 in mediating polymicrobial interactions during periodontal disease. I agree that the manuscript presents an interesting mechanistic study addressing an important question in host-pathogen interactions and that the combination of in vitro and in vivo approaches represents a strength of the work.

At the same time, I believe that several aspects should be addressed to strengthen the manuscript and to better position it for publication in PLOS Pathogens. In particular, I ask that you carefully consider the following points during revision.

Major points

1. Mechanistic interpretation and causal relationships: While the data support an important role for NLRP6 in the observed phenotype, the proposed mechanistic pathway linking P. gingivalis-induced macrophage polarization, enhanced bacterial persistence, NLRP6 activation, and increased IL-1β production is not fully established by the current data. If additional experimental evidence supporting this mechanistic model is available, it would considerably strengthen the manuscript. Otherwise, I ask that you revise the text to more clearly distinguish experimentally demonstrated findings from mechanistic interpretations and explicitly discuss these limitations.

2. Generalizability of the findings: The study relies on single strains of both P. gingivalis and S. gordonii. Although I do not consider this a major limitation of the study, the manuscript would benefit from a discussion of how strain-to-strain variability or the use of other periodontal pathogens might influence the broader applicability of the findings.

3. Physiological relevance of the experimental infection conditions:  The infection model employs a multiplicity of infection that is commonly used. However, it would be helpful to discuss the physiological relevance of these conditions. If similar observations were obtained using lower bacterial inocula, please include this information. Otherwise, please address this point in the Discussion.

4. Discussion and interpretation of the in vivo findings: I encourage you to further strengthen the Discussion by placing the findings into the broader context of polymicrobial host-pathogen interactions and innate immune regulation in periodontal disease. In addition, please ensure that the conclusions drawn from the ligature model are appropriately balanced, particularly if bacterial challenge does not significantly increase bone loss beyond that observed with ligature alone.

In addition to these editorial comments, please carefully address each of the reviewer’s comments in a detailed point-by-point response. Several of the reviewer’s suggestions relate to clarification of statistical analyses, data presentation, figure interpretation, and methodological details, and I believe addressing these comments will further improve the manuscript.

I look forward to receiving your revised manuscript and detailed response to the comments raised during review.

**Journal Requirements:**

If the reviewer comments include a recommendation to cite specific previously published works, please review and evaluate these publications to determine whether they are relevant and should be cited. There is no requirement to cite these works unless the editor has indicated otherwise.

**Reviewers' Comments:**

Reviewer's Responses to Questions

**Part I - Summary**

Please use this section to discuss strengths/weaknesses of study, novelty/significance, general execution and scholarship.

Reviewer #1: This is a significant study in which the authors investigate the mechanistic basis of synergistic pathogenicity in periodontal disease. P. gingivalis was shown to stimulate an inflammatory phenotype in macrophages which facilitated survival of S. gordonii. In turn S. gordonii stimulated the NLRP6 inflammasome resulting in increased production of IL-1β. In an in vivo model of periodontal disease, NLRP6 ko mice show less inflammation and bone loss following P. gingivalis challenge. These results provide novel insights into the pathogenicity of P. gingivalis and its dependence on the presence of co-infecting bacteria. Additionally, NLRP6 is established as an important node in the inflammatory pathways that contribute to tissue destruction.

**********

**Part II – Major Issues: Key Experiments Required for Acceptance**

Please use this section to detail the key new experiments or modifications of existing experiments that should be absolutely required to validate study conclusions.

Generally, there should be no more than 3 such required experiments or major modifications for a "Major Revision" recommendation. If more than 3 experiments are necessary to validate the study conclusions, then you are encouraged to recommend "Reject".

Reviewer #1: (No Response)

**********

**Part III – Minor Issues: Editorial and Data Presentation Modifications**

Please use this section for editorial suggestions as well as relatively minor modifications of existing data that would enhance clarity.

Reviewer #1: 1. Data in Fig1 should include a statistical analysis

2. Fig 2 D, E, F, provide some explanation for the different pattern with the RAW264.7 cells

3. Fig 3C. Is there a statistical difference compared to S. gordonii alone, this should be shown, as the text indicates that it is S. gordonii that induces IL-1β

4. Fig 3 A-C. For completeness, experiments should be run with P. gingivalis and S. gordonii alone at numbers equivalent to those used in the mixtures

5. Discuss if the effect of P. gingivalis is specific to this organism or would other periodontal pathogens show the same phenotype

6. Fig 4C. A quantitative analysis of co-localization should be performed

7. Fig 5B. There appears to be no statistical difference in bone loss in WT between ligature alone and ligature + P. gingivalis. This indicates that bacteria are not the primary driver of bone loss in this model, so results should be interpreted more cautiously.

8. Line 325 and elsewhere. Please describe how the WT and ko mice were ‘matched’

**********

PLOS authors have the option to publish the peer review history of their article (what does this mean?). If published, this will include your full peer review and any attached files.

If you choose “no”, your identity will remain anonymous but your review may still be made public.

**Do you want your identity to be public for this peer review?**  For information about this choice, including consent withdrawal, please see our Privacy Policy.

Reviewer #1: No

[NOTE: If reviewer comments were submitted as an attachment file, they will be attached to this email and accessible via the submission site. Please log into your account, locate the manuscript record, and check for the action link "View Attachments". If this link does not appear, there are no attachment files.]

**Figure resubmission:**

**Reproducibility:**

?>

# Author comment: Author response to Decision Letter 1

**DOI:** 10.1371/journal.ppat.1014574.r002

31 Jul 2026

Attachment Submitted filename: Response to reviewers.pdf

# Editor report: Decision Letter 1

**DOI:** 10.1371/journal.ppat.1014574.r003

**Authors:** Michael Wessels, Anders Hakansson

25 Aug 2026

Dear Dr. Kay,

We are pleased to inform that your manuscript, "Polymicrobial-driven NLRP6 inflammasome regulates IL-1β production and alveolar bone loss in a murine model of periodontitis", has been editorially accepted for publication at PLOS Pathogens.

Before your manuscript can be formally accepted and sent to production, you will need to complete our formatting changes, which you will receive by email within a week. Please note that your manuscript will not be scheduled for publication until you have made the required changes.

IMPORTANT NOTES

(1) Copyediting and Proofreading: The corresponding author will receive a typeset proof for review, to ensure errors have not been introduced during production. Please review the PDF proof of your manuscript carefully, as this is the last chance to correct any errors. Please note that major changes, or those which affect the scientific understanding of the work, will likely cause delays to the publication date of your manuscript.

(2) Appropriate Figure Files: Please remove all name and figure # text from your figure files. Please also take this time to check that your figures are of high resolution, which will improve the readbility of your figures and help expedite your manuscript's publication. Please note that figures must have been originally created at 300dpi or higher. Do not manually increase the resolution of your files. For instructions on how to properly obtain high quality images, please review our Figure Guidelines, with examples at: http://journals.plos.org/plospathogens/s/figures.

We encourage you to use PACE (http://pace.apexcovantage.com/), a figure-checking tool which helps authors ensure all figures meet PLOS requirements so that figures can publish in the highest quality. PACE is a digital diagnostic and conversion tool for figure files. It will provide information about any failed check(s) and, if able, will automatically convert the figure into an acceptable file that passes quality checks.

To use PACE, you must first register as a user. Then, login and navigate to the UPLOAD tab, where you will find detailed instructions on how to use the tool.

(3) Striking Image: Please upload a striking still image to accompany your article if one is available (you can include a new image or an existing one from within your manuscript). Should your paper be accepted, this image will be considered for our monthly issue image and may also appear on our website to feature your article. Please upload this as a separate file, selecting "striking image" as the file type upon upload. Please also include a separate "Other" file with a caption, including credits and any potential copyright information. Please do not include the caption in the main article file. If your image is from someone other than yourself, please ensure that the artist has read and agreed to the terms and conditions of the Creative Commons Attribution License at http://journals.plos.org/plospathogens/s/content-license. Please note that PLOS cannot publish copyrighted images.

(4) Press Release or Related Media: If you or your institution will be preparing press materials for this manuscript, please inform our press team in advance at plospathogens@plos.org as soon as possible. We ask that you contact us within one week to plan ahead of our fast Production schedule. If you need to know your paper's publication date for related media purposes, you must coordinate with our press team, and your manuscript will remain under a strict press embargo until the publication date and time.

(5) Update your Profile Information: Now that your manuscript has been provisionally accepted, please log into Editorial Manager and update your profile, if needed. Go to http://www.editorialmanager.com/ppathogens, log in, and click on the "Update My Information" link at the top of the page. Please update your user information to ensure an efficient production and billing process.

(6) LaTeX users only: Our staff will ask you to upload a TEX file in addition to the PDF before the paper can be sent to typesetting, so please carefully review our Latex Guidelines http://journals.plos.org/plospathogens/s/latex in the meantime.

Thank you again for supporting Open Access publishing; we are looking forward to publishing your work in PLOS Pathogens.

Best regards,

Anders P Hakansson, Ph.D.

Academic Editor

PLOS Pathogens

Michael Wessels

Section Editor

PLOS Pathogens

Dominique Soldati-Favre

Editor-in-Chief

PLOS Pathogens

***********************************************************

# Editor report: Acceptance letter

**DOI:** 10.1371/journal.ppat.1014574.r004

**Authors:** Michael Wessels, Anders Hakansson

Dear Dr. Kay,

We are delighted to inform you that your manuscript, "Polymicrobial-driven NLRP6 inflammasome regulates IL-1β production and alveolar bone loss in a murine model of periodontitis," has been formally accepted for publication in PLOS Pathogens.

We have now passed your article onto the PLOS Production Department who will complete the rest of the pre-publication process. All authors will receive a confirmation email upon publication.

Thank you again for supporting open-access publishing; we are looking forward to publishing your work in PLOS Pathogens.

Best regards,

Dominique Soldati-Favre

Editor-in-Chief

PLOS Pathogens
